# Proof of Principle: Is a Pre-treatment Behavior Approach Test a Potential Predictor for Response to Intensive Residential Treatment in Patients With Treatment Refractory Obsessive Compulsive Disorder?

**DOI:** 10.3389/fpsyt.2021.662069

**Published:** 2021-07-21

**Authors:** Malinda van Geijtenbeek-de Vos van Steenwijk, Aart de Leeuw, Harold van Megen, Jonathan Selier, Henny Visser

**Affiliations:** ^1^Marina de Wolfcentrum, Centrum Voor Psychotherapie, GGZ Centraal, Ermelo, Netherlands; ^2^Academisch Angstcentrum, Altrecht, Utrecht, Netherlands

**Keywords:** obsessive-compulsive disorder, treatment refractory, intensive residential treatment, Behavior Approach Test, willingness, exposure response prevention therapy, cognitive behavior therapy

## Abstract

Patients with severe and treatment refractory obsessive compulsive disorder (OCD) are usually referred to a specialized center for intensive residential treatment (IRT), consisting of exposure and response prevention (EX/RP), pharmacotherapy and additional therapies. About 50% of the patients does not respond to IRT. Currently we are not able to predict treatment response. If we were to have predictive tools, we could personify treatment at an earlier stage. Recent studies show that early adherence and willingness to EX/RP and low avoidance during EX/RP measured during treatment were associated with treatment response. In this observational study willingness and ability of patients with severe and treatment refractory OCD (*N* = 58) is conceptualized by a behavioral measurement, measured before the start of 12 weeks of IRT, using a Behavior Approach Test (BAT), as opposed to relying on self-report measurements. A medium or strong association between pre-treatment performance on the BAT and treatment response would justify next steps to test the BAT as a predictive tool for IRT. Results of regression analyses showed that there is a significant association between the performance on the BAT and change in OCD symptom severity after IRT. However, the effect-size is too small to use the BAT in its current form as predictor in clinical practice. The principle of the association between pre-treatment behaviorally measured willingness and ability to fully engage in EX/RP, and treatment response has now been proven. To ultimately design a predictive tool, future research is needed to refine a behavioral measurement of pre-treatment willingness and ability.

## Introduction

Obsessive compulsive disorder (OCD) is a serious, disabling and often chronic psychiatric disorder, characterized by obsessive thoughts and compulsive behavior (1, 2).

Cognitive behavioral therapy (CBT) and pharmacotherapy with serotonin reuptake inhibitors (SSRI) are effective treatments for OCD (3–5). About 50–60% of the patients respond to these treatments (6, 7). The next step in the algorithm for non- or partial responders, according to the internationally used UK multidisciplinary treatment guidelines, is an “intensive treatment and inpatient service.” The treatment and service are not further specified (8, 9). Usually it consists of CBT with daily therapist guided exposure with response prevention (EX/RP), cognitive (group-) therapy, additional pharmacotherapy and treatment modules, such as non-verbal treatment and family treatment. This intensive residential treatment (IRT) usually takes place in a residential or day-clinical specialized OCD treatment center (10).

Recent studies found IRT to be effective for severe or treatment refractory OCD. About 50–60% of the patients with remaining severe OCD symptoms after outpatient CBT and SSRI, do benefit from IRT (10–13). This suggests that the other half does not and these high rates of non-response urge us to enhance and further personify treatment for this patient group.

Studies have attempted to identify factors to predict treatment response for outpatient treatments. Findings were contradictory (6). Olantunji and colleagues conclude in their meta-analysis that the study-design was often not fit to test the predictive value and suggest to use prospective designs to learn more about these phenomena.

Until recently little was known about predictors for treatment response among patients with severe and treatment refractory OCD after IRT.

In the last 15 years, several studies were conducted to close this gap. The only systematic review and meta-analysis conducted on this subject (10) and published in 2016 found that marital status was often replicated as a predictor (5 out of 6 studies) as was the severity of OCD at admission (5 out of 8 studies), but overall there were no consistent predictors for treatment outcome. Interestingly all reviewed studies focused mostly on sociodemographic characteristics, co-morbidity and severity of OCD as potential predictors. Some more recent studies kept this focus and found additional evidence for severity as a predictor for non-response to IRT, specifically, that severity of obsessions was associated with poorer treatment outcomes (12) and for poor insight (little to no acknowledgment of the irrational nature of OCD symptoms) (14).

Other recent studies focused less on sociodemographic characteristics and severity and researched other promising concepts: low behavioral avoidance during EX/RP (15), early adherence to EX/RP tasks during treatment (16) and verbalized willingness to the EX/RP during treatment (17). They were found as predictor in studies among patients with severe and refractory OCD and among patients with moderate OCD. “Willingness” was assessed by a short questionnaire, about the willingness to fully experience unpleasant and unwanted thoughts, emotions and bodily sensations during exposure, and was found to be associated with faster symptom reduction during IRT. Another examined concept is readiness to exposure. This was assessed by a 3 item pre-treatment questionnaire and predicted better adherence to EX/RP. Its predictive value for treatment outcome was not examined (18, 19). Clinical experience and previous research in other patient groups such as patients with phobia do however suggest that low adherence to EX/RP and avoiding feared situations during treatment are important factors in non-response to EX/RP (16, 20–22).

Based on these findings, we expect that information concerning the extent to which a patient is able and willing to fully engage in EX/RP, is associated with treatment outcome. It stands to reason that if one is willing and able to expose oneself to ones feared situations at the start of a treatment, one will also be inclined to do so during treatment with high patient adherence, resulting in a better response to the treatment.

The aim of this study is to examine the association between pre-treatment performance on a behavioral test on willingness and ability to fully engage in EX/RP and response to IRT. We developed a behavioral measurement, the Behavior Approach Test (BAT), adaptable to heterogenic OCD symptoms. A medium or strong association, as reflected by a cohen's *f* ^2^ ≥ 0.15 between pre-treatment performance on the BAT and treatment response would be clinically significant and justify to test the predictive value of the BAT in future research. This can ultimately contribute to the development of a go-no go test for IRT or an instrument that may contribute to personifying treatment for patients with severe, treatment refractory OCD.

We hypothesized that there is a clinically significant association between the pre-treatment BAT-score and symptom change in OCD after 12 weeks of IRT is to examine this principle on its feasibility to ultimately be able to predict treatment outcome for complex, treatment refractory OCD after IRT on base of a pre-treatment test on willingness and ability.

## Materials and Methods

### Design

We used an observational cohort study-design. Patients were informed and asked for prior consent to participate to the study. No changes were made to the trial design after the start.

### Participants

The study was performed at the Marina de Wolf Centrum, Centrum voor Psychotherapie of GGZ Centraal, a supra-regional specialized OCD treatment center in the Netherlands. All patients with OCD who were referred for IRT to this treatment center, were asked to participate in the study. All participants had a history of regular treatment, in accordance with the Dutch multidisciplinary guidelines (CBT and at least 1 adequately dosed SSRI trial) (8).

After the regular intake procedure, participants were informed about the study, and gave informed consent. They were told that the aim of the study was to find out whether the way people perform on exposure exercises during the BAT can predict treatment outcome of IRT.

Patients were eligible to participate in the study if they: (1) were aged 18 years or older, (2) met a primary DSM 5 diagnosis of OCD, (3) were referred for IRT, and (4) gave an informed consent. OCD diagnosis was established by Mini-Schedules for Clinical Assessment in Neuropsychiatry (Mini-SCAN) (23, 24).

The exclusion criteria were: (1) a primary psychotic disorder, (2) an organic mental disorder, (3) a severe substance dependence, (4) intellectual disability, or (5) an insufficient command of the Dutch language.

### Measurements

#### BAT

The level of willingness and ability to engage in EX/RP was measured by a Behavior Approach Test (BAT), which was specially designed for this study. In this BAT, a participant is able to demonstrate the pre-treatment ability and willingness to fully engage in EX/RP. The BAT consists of a 1-h pre-treatment exposure session in which a participant is asked to take as many steps as possible on an idiosyncratic hierarchy of exposure tasks, ascending in difficulty.

The procedure is as follows: At the center, the treatment as usual starts with an outpatient diagnostic phase prior to the start of the IRT. In this diagnostic phase all patients in collaboration with a CBT therapist set up a list of their primary OCD symptoms for which they seek treatment. Multiple feared situations, which the patient avoids or only approaches while performing compulsions are identified. Based on this a range of corresponding exposure tasks are set up, in which the patients can expose themselves to the specific feared situations while refraining from neutralizing behavior. This list is then used throughout the IRT.

Specifically for the BAT, the participant and therapist selected 10 tasks from this list based on the expected anxiety when performing the task, ranked in equally ascending steps from 1 (hardly any distress expected) to 10 (maximum distress expected). All 10 tasks had to be completed within 1 h. As part of the BAT, the participant and therapist also set up instructions on how the participant would abstain from possible neutralizing behavior or rituals after finishing the BAT (e.g., not cleaning the house afterwards for at least 24 h). The BAT could be performed at several locations, if relevant for the specific exercises (mostly at home, but also in a shop, public bathroom, etc.).

The participants were instructed to perform the exposure tasks described on the list, starting with step 1 and trying to go as far as they could up to step 10. Every 2 min subjective units of distress (SUD) (0–10) (25) were established. The participant decided when to stop. Although they were firmly encouraged to take as many steps as possible on the BAT, there were no consequences for the number of correctly conducted steps. A CBT-educated psychiatric nurse, familiar with this specific patient-population and trained in the BAT-procedure, guided the BAT. They recorded the SUDs, registered whether steps were correctly conducted, and videotaped the BAT for assessors.

The BAT-score is the number of successfully performed succeeding steps on the BAT (range 0–10). Independent assessors scored the number of steps the participant had taken correctly on the BAT, by comparing the description of each step on the idiosyncratic BAT list with the video-taped behavior of the participant. In case of any possible ambiguity concerning the correctness of the taken steps or presence of compulsions, avoidance or rituals, or when their score did not correspond with the rating of the guiding nurse, a second rating was done by another assessor and a compromise was made between both assessors. Assessors were CBT-educated, mental healthcare professionals, familiar with this specific patient-population.

#### Severity of OCD

Severity of OCD-symptoms was assessed using the Yale Brown Obsessive Compulsive Scale (YBOCS). The YBOCS is a semi-structured interview and consists of 10 items with a 0–4 scale. The total score ranges from 0 to 40. Higher scores indicate greater severity of the OCD. This is a reliable and valid instrument and the golden standard for measuring OCD-severity (26). Cronbach's α for this scale is 0.80.

Conform international expert consensus responder status was defined as a decrease of the YBOCS score between the beginning and end of the treatment of at least 35%. Remission status was defined by a YBOCS score of ≤12 (27).

#### Duration and Chronicity of OCD

The duration of OCD was assessed based on a self-report questionnaire (in years).

Chronicity of OCD was assessed through a self-report questionnaire. Patients were asked whether they had continuously experienced at least moderate severe OCD over the past 2 years (1).

#### Comorbidity

Comorbidity was assessed by Mini-Schedules for Clinical Assessment in Neuropsychiatry (Mini-SCAN) and Structured Clinical Interview for Mental Disorders II (SCID II). These instruments are designed to objectively and in a structured way classify disorders based on the criteria of the DSM 5 (23, 24, 28). The presence of a comorbid autism spectrum disorder was assessed based on the hospital file, taking the current guidelines for diagnosing autism into account (29).

The assessment was conducted at beginning of the treatment (week 0) and the outcome measurement was taken at the beginning (week 0) and end of the treatment (week 12).

### Blinding

The treatment team did not know the BAT-score. The assessors and the raters of the BAT-videos were not part of the treatment team and therefore did not know the patients treatment-course nor the outcome.

### Training

The assessors and the nurses guiding the BAT were trained, monitored, and supervised in the rating and assessment techniques.

### Power Considerations

To calculate what effect sizes could reliably be detected with the included number of participants, a sensitivity analysis for linear multiple regression analysis, Fixed model, *R*^2^ Increase, with one tested predictor (total number of predictors: 2) was performed using G-Power 3.1.9.7. (30). With an alpha set to 0.05 and a beta of 0.2 (power of 80%), the current sample size *n* = 58 was sensitive to detect medium size effect of BAT score on treatment outcome (*f* ^2^ = 0.14).

### Statistical Analysis

Characteristics of the participants, the BAT-scores and the response to treatment were summarized using descriptive statistics. To compare patients who refused participation with participants on baseline OCD severity and symptom change after 12 weeks of IRT, two independent-samples *T*-test were performed.

To determine the explained variance of the BAT for symptom change, adjusted for baseline OCD severity, first baseline OCD severity was entered in a multivariate regression analysis and second the BAT-score.

A possible interaction effect was considered between baseline OCD severity and the BAT-score. Therefor an interaction variable “OCD severity x the BAT-score” was constructed and a separate multivariate regression analysis was performed. This was done by firstly entering baseline OCD severity, secondly the BAT-score and thirdly the interaction variable “OCD severity x the BAT-score.” To adjust for collinearity between baseline OCD severity and symptom change the variables were centered before being entered to the analyses. Possible violations of the assumptions of normality, linearity and homoscedasticity were checked. To test for multicollinearity the variance inflation factor and tolerance were calculated.

Statistical calculations were performed using the Statistical Package for Social Sciences version 25. All *p-*values were two-tailed and statistical significance was set at *p* < 0.05.

### Ethics

The design and conduct of the study were approved by the medical ethics review board METc VUmc (Amsterdam, the Netherlands).

## Results

### Participants

From January 2017 until October 2019 83 patients, met the inclusion criteria and were invited to participate. Three patients met the exclusion criteria and 19 patients refused to participate. They were asked for consent for the use of other personal information for this study, such as baseline OCD severity and symptom change after 12 weeks IRT. Nine of these 19 patients gave that consent. From 7 of these non-participants we had outcome measures to our disposal.

**Figure 1:**
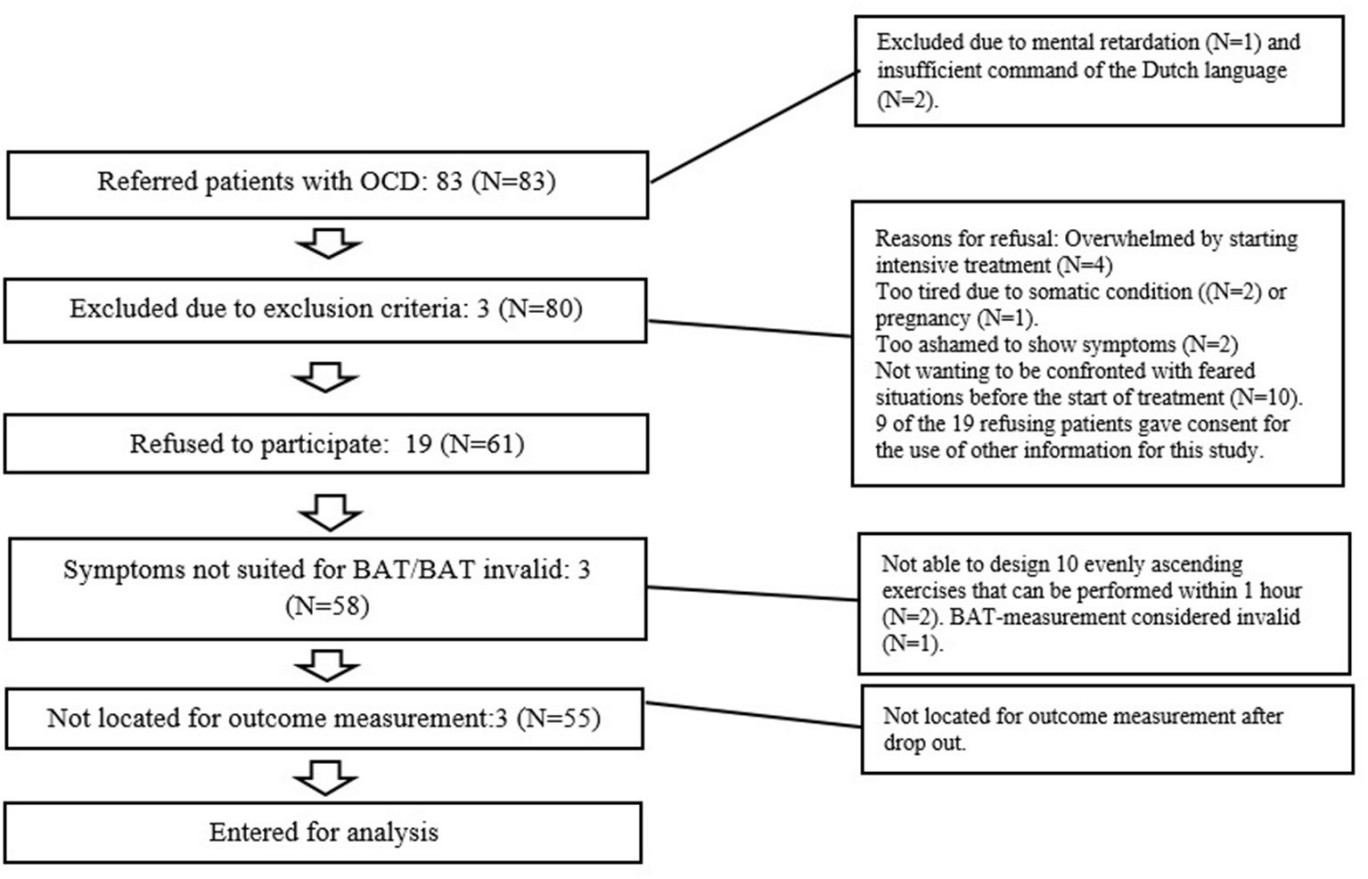
Participant-inclusion from referral to analysis.

Two patients of the remaining 61 patients could not participate due to their specific type of obsessions and compulsions, which were not suitable for exposure in the BAT-format (10 exposure tasks that can be performed within 1 h). For one participant consensus was reached to exclude the measurement, due to the patients' personal crisis-like circumstances (not related to the BAT) that occurred the day the BAT was performed, which rendered the measurement to be invalid. This left 58 patients to be included in the study. From the 4 participants that stopped with the therapy prematurely, 3 participants could not be located for the outcome measurement (see Figure 1).

There were 27 male (47%) and 31 female (53%) participants with an average age of 32.9 years (SD = 14.5). On average they had a severe level of OCD, as reflected by a mean score of 28.7 (SD = 5.1) on the YBOCS. The average age of onset of OCD was 20.5 year (SD = 9.1) and the average duration of symptoms before entering the study was 11.5 years (SD = 13.3). Fifty-one participants (88%) had chronic OCD. Nearly all participants (56 participants, 97%) had one or more comorbid disorders. Forty participants (69%) had a trait disorder (personality disorder and/or autism spectrum disorder).

After 12 weeks of IRT 29 participants (53%) responded to the therapy. For 11 patients (20%) the OCD was in remission, reflected in a YBOCS score ≤ 12. There was an average improvement of 11.0 (SD = 8.0) points on the YBOCS. At the end of the treatment participants had on average a moderate level of OCD, as reflected by a mean score of 17.5 (SD = 7.6). Four participants (7%) stopped with the therapy prematurely (see Table 1).

**Table 1:** Demographical and clinical characteristics of the sample.

**Variable (*N* = 58)**	**Mean (SD)/*N* (%)**
Demographical characteristics	
Male, %	27 (47%)
Female, %	31 (53%)
Age (years)	32.9 (14.5)
OCD	
Severity (YBOCS score)	28,7 (5.1)
Age of onset (years)	20.5 (9.1)
Duration of symptoms (years)	11.5 (13.3)
Chronic OCD (yes)	51 (88%)
Comorbidity next to OCD	56 (97%)
Comorbidity state^a^	
Number of comorbid state disorders^a^	1.3 (0.9)
One or more mood disorder(s) (yes)	39 (72%)
One or more anxiety disorder(s)	21 (39%)
somatic symptom disorder (yes)	2 (4%)
Substance use disorder (yes)	4 (7%)
Comorbidity trait^b^	
Presence of trait-disorders^b^	40 (69%)
One or more personality disorder(s) (yes)	32 (55%)
Autism spectrum disorder (yes)	10 (17%)
**Symptom change after 12 weeks of IRT**	
OCD severity after 12 weeks (YBOCS)	17.5 (7.6)
Symptom change (ΔYBOCS)	11.0 (8.0)
Responders (YBOCS > 35% reduction, yes)	29 (53%)
Remission (YBOCS ≤ 12, yes)	11 (20%)
Stopped therapy prematurely	4 (7%)

a *Comorbidity state: Presence of comorbid disorders assessed through Miniscan, former Axis I disorders conform DSM IV*.

b *Comorbidity trait: Presence of one or more personality disorder(s) and/or an autism spectrum disorder*.

An independent-samples *t*-test was conducted to compare the baseline OCD-severity for participants that chose to participate and for patients who chose not to. There was no significant difference in YBOCS-scores of the participants (*N* = 58) (M = 28.7, SD = 5.1) and the non-participants (*N* = 9) [M = 30, SD = 3.3; *t*_(66)_ = −0.73, *p* = 0.47]. Another independent-samples *t*-test was conducted to compare symptom change for participants of the study and for patients who chose not to participate. There was no significant difference in symptom change of the participants (*N* = 55) (M = 11.3, SD = 1.1) and the non-participants (*N* = 7) [M = 11.0, SD = 3.3; *t*_(61)_ = 0.08, *p* = 0.94]. We were not able to locate 3 participants for the outcome measurement after they prematurely dropped out of treatment. They were therefore excluded from the outcome analyses (see Figure 1).

### BAT

Fifty-eight BAT's were designed, performed and considered valid.

Participants took an average of 7.8 steps, range 1–10 (SD = 2.7). Forty-two percentage of the participants reached the last step (step 10) on their BAT (see Table 2). The highest rating of the SUD during the BAT was on average 8.2, range 4–10 (SD = 1.6).

**Table 2:** BAT-score at start of treatment.

**BAT (*N* = 58)**	***N* (%)**
Highest step at	
Step 0 (no successful steps)	0
Step 1	2 (3%)
Step 2	0 (0%)
Step 3	2 (3%)
Step 4	4 (7%)
Step 5	4 (7%)
Step 6	1 (2%)
Step 7	5 (9%)
Step 8	8 (14%)
Step 9	7 (12%)
Step 10	25 (42%)

### Relation Between the BAT-Score and Symptom Change

There were no violations of the assumptions of normality, linearity and homoscedasticity and collinearity diagnostics revealed that multicollinearity was not a problem for the analyzed models.

The results of the hierarchical multivariate regression analysis showed that the first model with only baseline OCD severity had a predictive value of 18%. After entry of the BAT-score (model 2), the total variance explained by the model as a whole was augmented with 6% to *R*^2^ = 24%, *F*_(1, 52)_ = 4.26, *p* < 0.05. The effect size of this 6% augmentation is an cohen's *f* ^2^ of 0.06, which is considered a small to medium effect size (see Table 3).

**Table 3:** Summary of the multivariate linear regression analysis for the relation between the BAT-score and symptom change.

	**Multivariate linear regression analysis with BAT-score, baseline OCD severity and symptom change**											
	**Model 1**^**a**^				**Model 2**^**b**^				**Model 3**^**c**^			
	**B**	**SE B**	***β***	***R***^**2**^	**B**	**SE B**	***β***	***R***^**2**^	**B**	**SE B**	***β***	***R***^**2**^
(Constant)	−7.34	5.54			−16.06	6.83			−12.35	7.21		
Severity OCD	0.73	0.19	0.42^**^		0.73	0.19	0.48^**^		0.68	0.19	−0.44^**^	
BAT-score					0.78	0.38	0.26^*^		0.55	0.40	0.18	
Interaction-variable									0.13	0.09	0.19	
*R*^2^				0.18				0.24				0.27
F for change in *R*^2^								4.26^*^				2.24

*Bold means significant with*

* *p < 0.05*;

** *p < 0.01*.

a *Predictors: (constant), baseline OCD severity*.

b *Predictors: (constant), BAT, baseline OCD severity*.

c *Predictors: (constant), BAT, baseline OCD severity and interaction-variable (BAT × baseline OCD severity)*.

When the effect of the baseline OCD severity is held constant, the YBOCS of a patient declines after 12 weeks of IRT by.78 point more with each extra step a participant takes during the pre-treatment BAT. The separate multivariate regression analysis, performed to examine a possible interaction effect, revealed there was no such effect.

## Conclusion and Discussion

This study examined the relation between the performance on a pre-treatment behavior approach test (BAT) and symptom change in treatment refractory patients with OCD after 12 weeks of Intensive Residential Treatment (IRT).

In line with our hypothesis, performance on the BAT was significantly associated with symptom change after IRT. Although statistically significant, the added value to the predictive value of baseline OCD severity alone is small: an augmentation from 18% to 24% predictive value for symptom change after 12 weeks of IRT. There was no significant interaction effect between BAT-score and baseline OCD severity.

The aim of this study is to examine the association between pre-treatment performance on a behavioral test on willingness and ability to fully engage in EX/RP (the BAT) and response to IRT. With a strong enough association (medium or strong) it would be warranted to test the predictive value of the BAT to ultimately contribute to the development of a go-no go test for IRT or an instrument that may contribute to personifying treatment for patients with severe, treatment refractory OCD.

For that ultimate goal the association between the BAT-score and symptom change ought to be at least medium. We conclude that although we found an association between the BAT-score and symptom change, its effect-size is too small to justify transforming the BAT in its current fashion into a clinically deployable instrument for indicating which treatment and treatment-setting is most promising for the individual patient. The statistical model including the BAT-score and baseline OCD severity predicts 24% of the symptom change, leaving 76% to not further specified factors. This leaves too much margin for error for a go-no-go test on an individual level.

This is to the best of our knowledge the first study examining the association between treatment outcome and pre-treatment willingness and ability to fully engage in EX/RP, by requesting a participant to actually carry out what they have verbally committed to.

Another strength of the study is the representativeness of the participants for the patient-group we aimed for in this study. Based on the clinical characteristics of the group of participants, we can conclude that it is a group of patients with chronic and severe symptoms and with predominantly one or more comorbid disorders, which were often personality or autism spectrum disorders. We attempt to improve treatment opportunities for specifically these patients and conducted this study to attribute to this goal.

Previous studies report more convincing evidence for (early) adherence and low avoidance as predictors for treatment response. One difference is that these studies included patients generally receiving first treatments in outpatient settings (15, 16). In the study of Reid and colleges, who examined willingness as a predictor for treatment outcome during IRT, participants surely had previous treatment, but as a condition only a history of pharmacological treatment was required for admission to the IRT (17). Perhaps patients with ongoing severe OCD after one or more adequately performed treatments with CBT, like the participants in our study, belong to a selective group, for whom other factors are more decisive for achieving a good treatment outcome. This possibly leaves a smaller role of importance for willingness and ability to EX/RP.

This being said, it should also be taken into account that there were some limitations that might have influenced our findings.

Firstly, although the participants were instructed and stimulated to take as many steps as possible, our participants were free to decide how far they would go, and -as it was part of a study- the reached BAT-score did not have consequences for their further treatment-course. A BAT fully integrated as a part of the assessment-procedure for the IRT will possibly have more impact.

Secondly the presence of the nurse and the element of being videotaped possibly influenced the way participants performed on the BAT in comparison to having to do the exposure tasks alone. If one or both of these possibilities are true, the BAT might not be a completely ecologically valid instrument to measure the willingness and ability to conduct EX/RP, possibly resulting in toned down findings.

Further, participants agreed to abstain from neutralizing behavior after finishing the BAT, however, it was not possible to check whether they really did not perform any of this behavior. This means that some BAT score might overestimate the level of engagement in EX/RP.

Also, the BAT may not have been challenging enough for certain patients. To our surprise it was observed that, when examining the number of steps taken by the participants on the BAT (Table 2), a large group (42.4%) reached step 10. In this sample the BAT was not able to further differentiate between the participants in their level of willingness and ability to expose themselves to their feared situations. Possibly the predictive value of the BAT will be greater, when a way is found to design some more challenging steps.

Another consideration is that the BAT tests were all idiosyncratically designed and focused on different subtypes of OCD, present in the sample. Thorough efforts were made by the therapists and patients to design BATs with between-patients comparable ascending steps from 1 to 10 to create a BAT-score that would resemble the level of willingness and ability to full EX/RP. However, every OCD is different, even within subtypes of OCD and therefore it is impossible to make the steps fully equal between for example a patient exposing oneself to say “forbidden” words and another patient exposing oneself to touch an uncleaned floor. Any attempt to design a naturalistic idiosyncratic instrument, relevant for the diverse clinical practice, carries the risk that for example step 4 of one BAT, might not completely comprise the same “amount” of willingness and ability to full exposure as step 4 on another BAT. If this is the case, this may have distorted the results to some extent.

Finally, possible bias may have emerged from the fact that a part of the patients that were eligible to participate refused to do so. Our impression was that for all 19 patients the great tendency to avoid exposure was a big factor for deciding to refuse to participate. We wonder whether this group would have had a low BAT-score. The BAT would possibly have more predictive value if this group could have been included. The comparisons we made between the participants and non-participants on baseline severity and symptom change do not give the impression there was a difference between the two groups. The small size of the group of non-participants of whom we could include their data in the analyses must be noted as limitation when interpreting these findings.

For further personifying treatments and maximizing therapeutic outcomes we are still in need of tools that can differentiate between the patients who do profit from IRT and the ones who do not or are in need of more extensive preparation trajectories. We suggest to further optimize the BAT to further examine the possible predictive value of these kind of tests.

We find it promising because—although little-there is an association between the BAT and symptom change after IRT, despite investigating this within this patient group with severe, chronic, complex and treatment refractory OCD, despite the fact that the BAT needs further fine-tuning to create more differentiation in the 42.4% that performed maximum on the current BAT, and despite the possible bias due to the non-participants.

Concluding, the predictive value of pre-treatment willingness and ability to fully engage in EX/RP does seem to be a promising field to further explore. We are still in great need of more instruments to predict treatment effect in patients with OCD, also for the patient group with severe, chronic and complex OCD. We consider our findings as a promising development in our quest to find one. More research is needed for a better understanding of the concept of willingness and ability to fully engage in EX/RP and its predictive value. This may help to further adapt or fine-tune the BAT in order to realize an effective predictive test for clinical use.

## Data Availability Statement

The raw data supporting the conclusions of this article will be made available by the authors, without undue reservation.

## Ethics Statement

The studies involving human participants were reviewed and approved by Medisch Ethische commissie, UMC Amsterdam, Locatie VUmc/De Boelelaan, Amsterdam, the Netherlands. The patients/participants provided their written informed consent to participate in this study.

## Author Contributions

MvG-dVvS, AL, HM, and HV contributed to conception and design of the study. MvG-dVvS, JS, and HV collected the data and organized the database. MvG-dVvS and HV performed the statistical analysis. MvG-dVvS wrote the first draft of the manuscript. All authors contributed to manuscript revision and approved the submitted version.

## Conflict of Interest

The authors declare that the research was conducted in the absence of any commercial or financial relationships that could be construed as a potential conflict of interest.

## Abbreviations

BAT: Behavior Approach Test
EX/RP: exposure and response prevention
IRT: intensive residential treatment.
